# Unusual Presentation of Chronic Hyperplastic Pulpitis: A Case Report

**Published:** 2008-01-10

**Authors:** Javad Faryabi, Shahrzad Adhami

**Affiliations:** 1 *Department of Oral and Maxillofacial Surgery, Faculty of Dentistry, Kerman University of Medical Sciences and Health Services, Kerman, Iran*; 2 *Department of Oral Pathology, Faculty of Dentistry, Kerman University of Medical Sciences and Health Services, Kerman, Iran*

**Keywords:** Case Reports, Dental Pulp Diseases

## Abstract

Chronic hyperplastic pulpitis (pulp polyps) usually occurs in molar teeth of children and young adults and is characterized by an overgrowth of granulomatous tissue into the carious cavity. Here, we report a rare type of pulp polyp in lower third molar of a 27-year-old woman that not only grow into carious cavity but also extruded in very large size that interfered with occluding of the teeth.

## INTRODUCTION

Hyperplastic pulpitis is a type of irreversible chronic pulpitis that occurs usually in young teeth where the pulp has been exposed by caries or trauma (1). Mechanical irritation and bacterial invasion result in a level of chronic inflammation that produces hyperplastic granulation tissue extrudes from the chamber and often fills the associated dentinal defect (2). In reviewing the literature we didn’t find that a pulp polyp may be enlarged to a size that resemble a large mushroom and occupies some space of oropharyngeal area, so we intended to report this unique case.

## CASE REPORT

**Figure 1:**
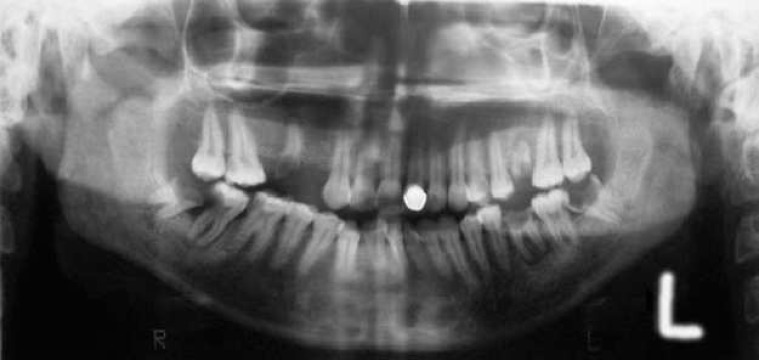
Left side semi impacted carious lower 3^rd^ molar was the origin of the lesion

**Figure 2:**
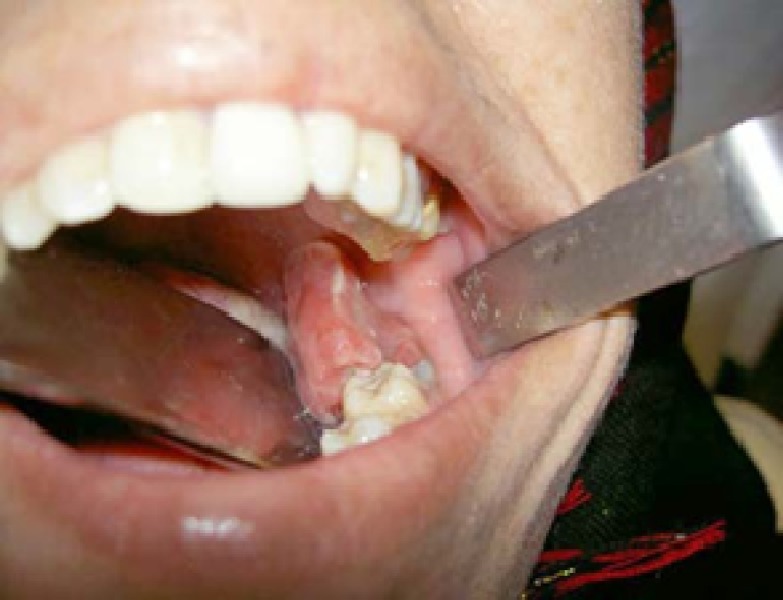
Unusual chronic hyperplastic pulpitis in unique large size

A 27-year-old woman referred for treatment of left side lesion of the oral cavity. She gave history of six months for its presence that enlarged gradually and interfered with eating and occluding the teeth, so that made patient worried about it (Figure 1), (Figure 2).

In review of systems (ROS) she gave history of idiopathic thrombocytopenic purpura and splenectomy of 12 years ago and caesarian section three years ago, but both of them had no relation to the present lesion. Laboratory examinations including CBC differential, WBC, platelet count, PT, and PTT were within normal limits, and in orthopanthogram radiography we observed left carious mesioangular semi impacted 3^rd^ molar with no specific lesion in bone and adjacent tissues (Figure 1).

Intraoral examination showed a large polypoid lesion with about 3.5 cm height, 1.5 cm width and a 5 mm stalk diameter protruded from the carious cavity of partially exposed crown of the tooth, the lesion overlaid on lingual side of this tooth and obviously presented itself in left oropharyngeal area.

So with considering the radiographic and clinical data these differential diagnoses were possible:

1. A large pulp polyp

2. Peripheral giant cell granuloma

3. Papilloma

The surgical procedure for excisional biopsy of the lesion and surgical removal of 3^rd^ molar tooth performed under local anesthesia (Persocaine-E, Darou Pakhsh-Tehran, Iran).

**Figure 3:**
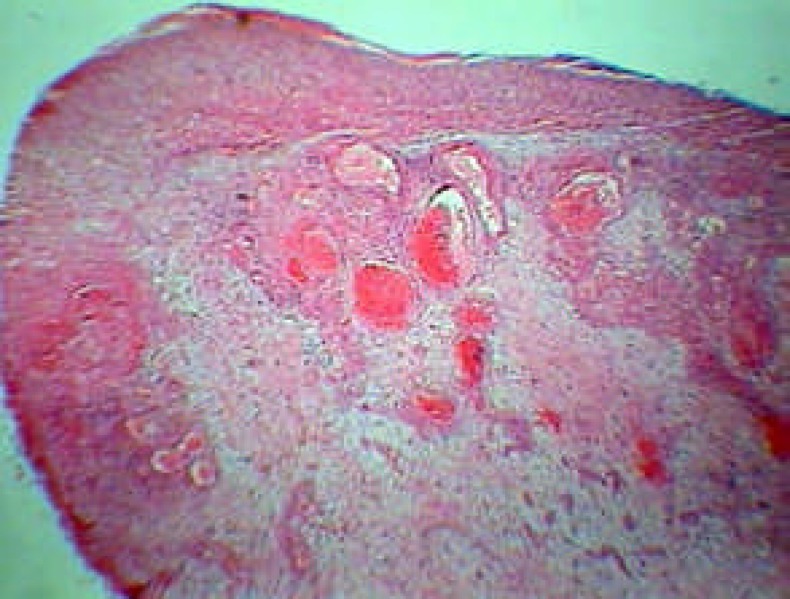
An exophytic mass of granulation like tissue (H&E; original magnification, ×100

We send the specimen included the carious 3^rd^ molar with base of the lesion in the carious cavity and the bulk of the lesion to the pathologist for histopathologic examination (Figure 3).

**Figure 4:**
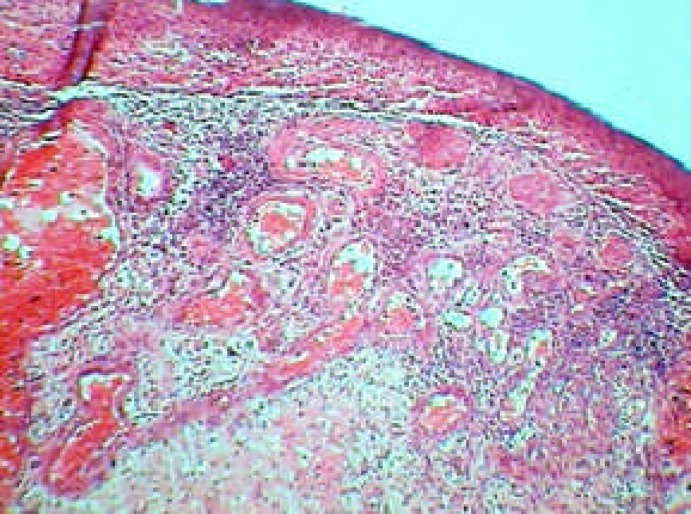
Specimen showing capillary blood vessels and a mixed inflammatory cell infiltrate of neutrophil, plasma cells and lymphocytes (H&E; original magnification, ×400)

**Figure 5:**
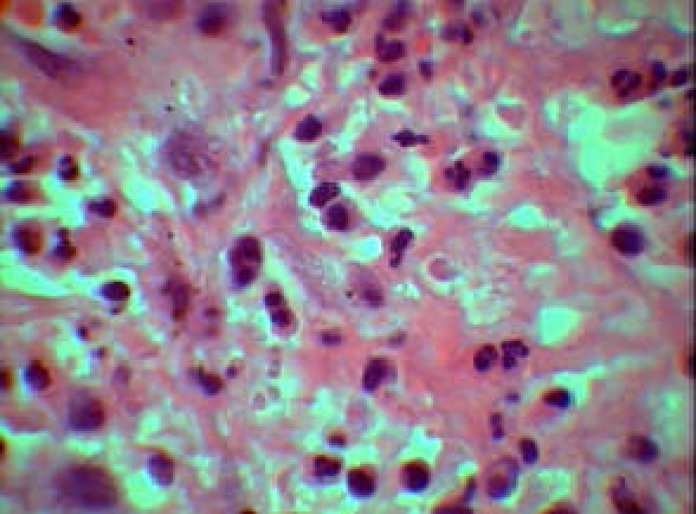
Mixed inflammatory cell infiltrate of neutrophil, plasma cells and lymphocytes (H&E; original magnification, ×1000)

## HISTOPATHOLOGICAL FINDINGS

Microscopic finding revealed a mass of inflamed granulation tissue resembling pyogenic granuloma that protruding from the. crown of the carious tooth. The fibrovascular stroma contained numerous capillary sized blood vessels lined by plump endothelial cells. There were also significant mixed inflammatory cells composed of lymphocytes, plasma cells and neutrophils.

The surface of the lesion was ulcerated and replaced by a fibrin purulent membrane.

Diagnosis was chronic hyperplastic pulpitis (Figure 4), (Figure 5).

## DISCUSSION

Hyperplastic pulpitis (pulp polyp) is the most visually dramatic of all pulp response, rising out of the carious shell of the crown and is a "mushroom" of living pulp tissue that is often firm and insensitive to touch (3).

It is a response of the pulp to acute inflammation occurs in young teeth but never in teeth of old patients, may this be indicative of a good pulpal response. Presumably the young pulp does not become necrotic following exposure, because its natural defenses and rich supply of blood allow it to resist bacterial infection. Transudates and exudates which are inflammatory response products in open chronic pulpitis, drain into the oral cavity and do not accumulate.

Thus intrapulpal pressure, which may consequently cause tissue damage and destruction of the microcirculation, does not develop (1).

According to chronologic time for development of the lower 3rd molar teeth between 4.4-22.1 years old (4-5), this patient had no young pulp in the beginning of formation of this lesion and we may consider rich blood supply to resist bacterial infection and slow process of carious formation due to partially impaction of crown of offending tooth led to developing the hyperplastic pulpitis in our patient.

The large size of the lesion in this patient may be due to:

**1. **No severe pain because this type of lesions usually has no pain and subside within seconds after the stimulus is removed (2).

**2. **No mobility of tooth and sensitivity to percussion because significant inflammation has not yet spread to the apical area (2).

**3. **Fear of surgical procedures and assume a lesion as a cancer (cancerophobia)

**4. **Neglected treatment by the patient for about six months

**5. **Good blood supply to the lesion

**6. **Continuous trauma from occluding the teeth.

## CONCLUSION

In treatment planning for Oral and maxillofacial lesions, we must consider the clinical finding, and dental history of the patient and finally the histopathologic report for management of such lesions in spite of unusual size of them.
